# A conserved inter-domain communication mechanism regulates the ATPase activity of the AAA-protein Drg1

**DOI:** 10.1038/srep44751

**Published:** 2017-03-17

**Authors:** Michael Prattes, Mathias Loibl, Gertrude Zisser, Daniel Luschnig, Lisa Kappel, Ingrid Rössler, Manuela Grassegger, Altijana Hromic, Elmar Krieger, Karl Gruber, Brigitte Pertschy, Helmut Bergler

**Affiliations:** 1 Institute of Molecular Biosciences, University of Graz, A-8010 Graz, Austria; 2 CMBI 260, NCMLS, Radboud University Nijmegen Medical Centre P. O. Box 9101, 6500HB Nijmegen, The Netherlands

## Abstract

AAA-ATPases fulfil essential roles in different cellular pathways and often act in form of hexameric complexes. Interaction with pathway-specific substrate and adaptor proteins recruits them to their targets and modulates their catalytic activity. This substrate dependent regulation of ATP hydrolysis in the AAA-domains is mediated by a non-catalytic N-terminal domain. The exact mechanisms that transmit the signal from the N-domain and coordinate the individual AAA-domains in the hexameric complex are still the topic of intensive research. Here, we present the characterization of a novel mutant variant of the eukaryotic AAA-ATPase Drg1 that shows dysregulation of ATPase activity and altered interaction with Rlp24, its substrate in ribosome biogenesis. This defective regulation is the consequence of amino acid exchanges at the interface between the regulatory N-domain and the adjacent D1 AAA-domain. The effects caused by these mutations strongly resemble those of pathological mutations of the AAA-ATPase p97 which cause the hereditary proteinopathy IBMPFD (inclusion body myopathy associated with Paget’s disease of the bone and frontotemporal dementia). Our results therefore suggest well conserved mechanisms of regulation between structurally, but not functionally related members of the AAA-family.

The protein family of AAA-ATPases (ATPases associated with a variety of cellular activities) achieves functional diversity by a shared core mechanism combined with pathway-specific adaptors^1^,^2^,^3^. Coordinated ATP hydrolysis in oligomeric complexes enables them to fulfil difficile cellular functions by unfolding of proteins and remodelling of protein complexes. Most AAA-ATPases form barrel-shaped hexameric rings, where each monomer consists of either one (type I) or two (type II) functional ATPase domains (named D1 and D2) accompanied by a regulatory N-terminal domain. The twelve ATPase domains present in a type II AAA-protein are arranged as two superimposed rings that can perform a rotary movement relative to each other^4^,^5^,^6^. To convert this motion into a vectored mechanical force required for substrate remodelling, it is crucial that the single domains act in a coordinated manner. The mechanisms that synchronize the individual sites are still under investigation and belong to the most discussed topics in the field^7^.

Well-studied models like the yeast ribosome biogenesis factor Drg1 or the multifunctional mammalian protein p97 have provided deep insights into the mechanisms of inter- and intramolecular synchronization of ATP binding and hydrolysis^7^,^8^,^9^. A central role in the regulation of activity can be assigned to the non-catalytic N-terminal domain that acts as connecting link between the enzymatic machinery and the target protein or protein-complex^10^,^11^. High resolution cryo-EM and crystal structures of p97 revealed that the position of the N-domain strongly depends on the nucleotide-binding states of the AAA-modules^5^,^12^,^13^,^14^,^15^; reviewed in^7^. In the ADP-bound state, the N-domain is found in the “down”-position co-planar to the D1 ring. In contrast, in the presence of the ATP-analogue ATPγS, the N-domain can adopt the “up”-conformation elevated above the D1 ring^12^,^14^,^16^. This conformational change was suggested to be a main regulatory switch for the ATPase activity of p97.

The N-domain of p97 mediates the direct physical interactions with the majority of co-factors and adaptor proteins that recruit the enzyme to the target pathways^7^,^10^,^17^. Adaptor-binding to the N-domain can either stimulate or inhibit the ATPase activity of p97 as exemplified by the adaptor proteins p37 and p47, respectively^18^. Beyond that, short linker segments between the individual domains serve as routes for the inter-domain signal transmission^14^,^19^. All these data indicate that the correct arrangement of the N-D1 segment (including the N-D1 linker) is crucial for regulation and intramolecular signal transmission in p97. This is further affirmed by the location of mutations in the p97 gene that cause the complex hereditary proteinopathy IBMPFD (inclusion body myopathy associated with Paget’s disease of the bone and frontotemporal dementia^20^,^21^,^22^) which is also being referred to as MSP1 (multisystem proteinopathy type 1^23^). These mutations cause amino acid substitutions at the N-D1 interface of the protein and affect the nucleotide-dependent positioning of the N-domain and the interaction with specific adaptor proteins^13^,^14^. Most p97 IBMPFD variants also exhibit increased basal (unstimulated) ATPase activity and for three of them (A232E, L198W, R155H) it was already shown that they additionally fail to be stimulated by the adaptor protein p37^11^,^13^,^18^,^24^. Structural characterization of pathological p97-IBMPFD variants revealed that these single amino acid exchanges at the N-D1 interface can also cause rearrangements of the distal C-terminal D2 AAA-domain and increase its hydrolytic activity^24^,^25^. This further strengthens the model of a regulatory link between the N-domain and both AAA-domains. The exact molecular basis of the detrimental effects caused by p97-IBMPFD mutations, however, is still a matter of debate. Recent NMR experiments with the p97 N-D1 fragment suggest that the position of the N-domain is in a dynamic, nucleotide-dependent equilibrium between the up- (ATP-dependent) and down-position (ADP-dependent)^26^. In IBMPFD variants this equilibrium is shifted towards the up-position.

Drg1 (Diazaborine resistance gene 1) is an essential type II AAA-ATPase in the yeast *Saccharomyces cerevisiae* and is highly related to mammalian p97 and its yeast orthologue Cdc48^27^. In previous works we have characterized Drg1 as a key player of eukaryotic ribosome biogenesis that initiates the final maturation of the large ribosomal 60S-subunit in the cytoplasm^8^,^28^,^29^. Drg1 binds to the C-terminal extension of the ribosome biogenesis factor Rlp24 (Ribosomal-like protein 24) and releases this shuttling protein from pre-60S particles shortly after their export from the nucleus^8^. This release is an essential prerequisite for all downstream maturation steps and therefore a crucial event in the highly regulated ribosome biogenesis pathway. Consequently, inactivation of Drg1 results in a failure to release and recycle Rlp24 as well as other shuttling proteins and export factors, also causing a block in early ribosome biogenesis steps^28^. The C-terminal extension of Rlp24 is not only responsible for the recruitment of Drg1 but also stimulates ATP hydrolysis in both AAA-domains^8^. The D2 domain is thereby essential for the energy dependent release of Rlp24 from the pre-ribosomal particle whereas the D1 domain modulates hexamerization and the interaction with Rlp24.

The AAA-domains of Drg1 and p97 show an amino acid sequence identity of about 50%, while the N-domains exhibit a significantly smaller degree of sequence conservation (Fig. S1). Nevertheless, fold recognition and secondary structure prediction indicate that the general architecture of the N-domain including the N-D1 interface are well conserved. In this study, we present novel mutations in the N-domain of Drg1 that cause a disturbed regulation of ATPase activity and altered nucleotide-dependent interaction with the substrate protein Rlp24. Concerning positioning within the protein and effects on basal and co-factor stimulated ATPase activity, these substitutions strikingly resemble the IBMPFD variants of p97. These findings suggest that exchanges at the N-D1 domain interface affect general properties of AAA-ATPases and indicate that the regulatory interplay between the N-domain and the two catalytic domains is shared by functionally unrelated members of this protein family.

## Results

### Mutations in the N-domain of *DRG1* cause a dominant negative growth phenotype

**Figure 1:**
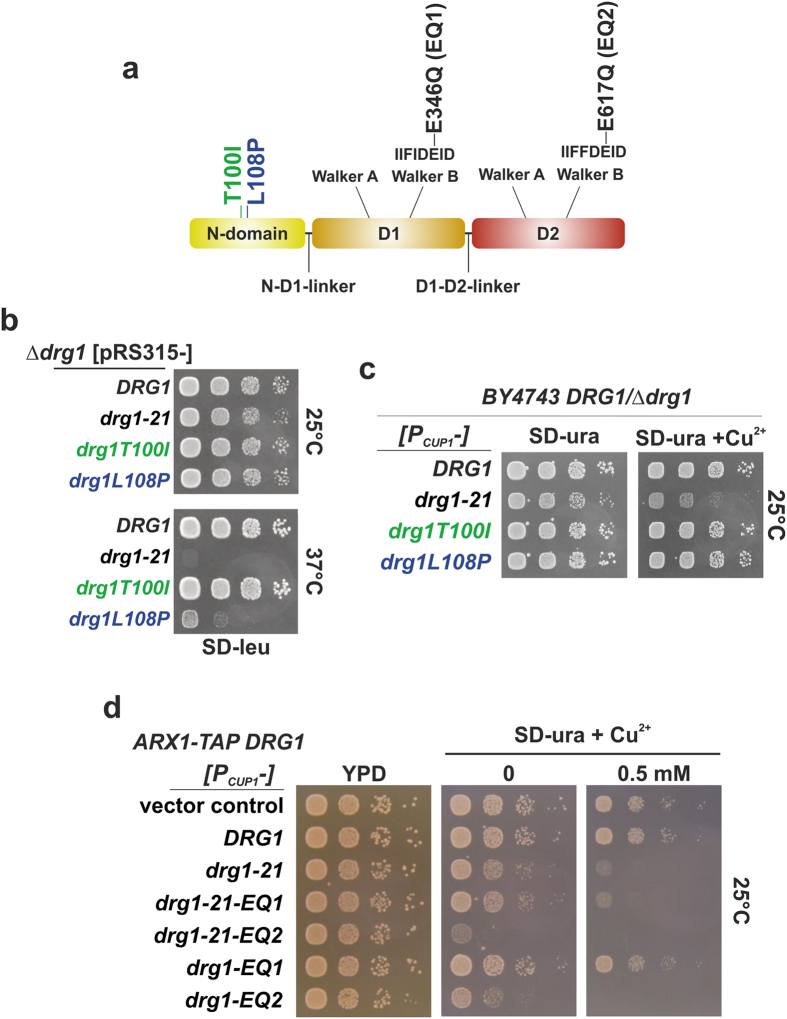
The *drg1-21* allele causes temperature sensitivity and a pronounced dominant negative growth phenotype. (**a**) Schematic domain representation of Drg1. The exchanges of the *drg1-21* allele are highlighted in green (T100I) and blue (L108P). (**b**) *Drg1* knockout strains carrying pRS315 (*LEU2*) plasmids expressing the *drg1-21* allele and alleles encoding the single exchanges (T100I and L108P) from their endogenous promoter were spotted on SD-leu and incubated at 25 °C and 37 °C. **(c)** The *drg1-21* allele and alleles encoding the single exchanges (T100I and L108P) were overexpressed from plasmids under the control of the inducible *CUP1* promoter (P_CUP1_) in the wild-type background. The cells were spotted on SD-ura as well as on plates containing 0.5 mM copper sulfate (SD-ura + Cu^2+^) to induce overexpression. (**d**) Different *DRG1* alleles were overexpressed from plasmids under the control of the inducible *CUP1* promoter (P_CUP1_) in the wild-type background. The cells were spotted on YPD, SD-ura and SD-ura + 0.5 mM copper sulfate to induce overexpression.

Random mutagenesis of *DRG1* and screening for mutations conferring temperature-sensitive growth phenotypes resulted in isolation of the novel *drg1-21* allele. This allele contains two point mutations causing two amino acid exchanges in the N-domain of Drg1; an exchange of threonine 100 for isoleucine (T100I) and of leucine 108 for proline (L108P), which together cause a pronounced growth defect at 37 °C (Fig. 1a,b). Individual analyses of these two substitutions showed that the L108P exchange was mainly responsible for the failure to grow at elevated temperature (Fig. 1b). In addition the L108P mutant showed a slight dominant negative growth defect upon overexpression from the copper inducible *CUP1* promoter in wildtype background (Fig. 1c). However, the full growth defect at 37 °C and a strong dominant negative effect were only observed when both exchanges were present, pointing at a synthetic enhancement of these two mutations. Interestingly, we found a similar dominant negative effect by overexpression of a Drg1 variant with inhibited ATP hydrolysis in the D2 AAA-domain (E617Q, designated Drg1-EQ2) but not for D1 (E346Q, designated Drg1-EQ1) as shown in Fig. 1d. In addition, the growth defect caused by the *drg1-21* allele was enhanced in combination with the EQ2 exchange, but not when combined with the EQ1 mutation. This is obvious on the SD-ura plate lacking copper where the *CUP1* promoter shows only low activity, but strong growth inhibition is observed for the allele containing the two *drg1-21* exchanges (T100I/L108P) in combination with the EQ2 exchange (Fig. 1d). This enhancement shows that the combination of both defects is extremely toxic for the cell indicating an intricate connection between the N-domain and the D2 domain of Drg1.

### Drg1-21 inhibits maturation of pre-60S particles

**Figure 2:**
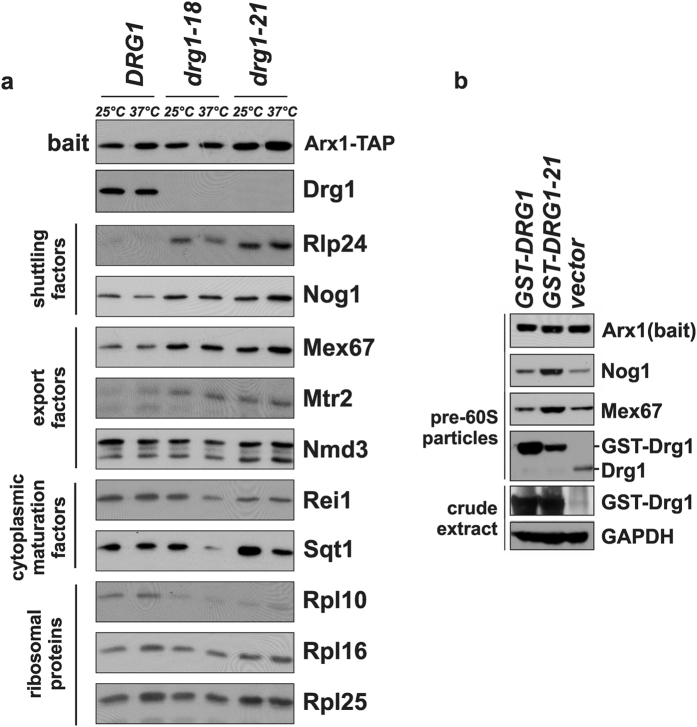
Drg1-21 is unable to perform its function in pre-60S particle maturation. (**a**) Drg1-21 shows reduced binding to pre-60s particles. Tandem affinity purifications of pre-60S particles using Arx1-TAP as bait protein were performed from strains expressing *drg1-21, drg1-18* or wildtype *DRG1* from their endogenous promoter. The protein composition of the purified particles was analysed by SDS-PAGE and western blotting with antibodies directed against the indicated factors. (**b**) Drg1-21 overexpression causes accumulation of shuttling proteins and export factors on pre-60S particles. Tandem affinity purifications of pre-ribosomal Arx1-TAP particles were performed from *DRG1* wildtype strains containing plasmids for overexpression (GST-Drg1 or GST-Drg1-21) or the empty vector control. Cells were grown to an OD_600_ of 1.0 (early log-phase) and overexpression of the GST-Drg1 variants from the *CUP1* promoter was induced by the addition of 0.5 mM CuSO_4_ and incubation for 3 hours. Original full-length images of cropped blots are provided in Supplementary Figs S7 and S8.

In order to assess the physiological implications of the exchanges leading to temperature sensitivity of the *drg1-21* allele we investigated binding of Drg1-21 to pre-ribosomal particles. For this purpose, we performed Tandem Affinity Purifications (TAP) using the shuttling pre-60S maturation factor Arx1 as bait protein (Fig. 2a). As controls, strains expressing either wildtype Drg1 or the temperature-sensitive Drg1-18 variant were used. Analysis of the protein composition of the purified Arx1-TAP particles revealed that Drg1-21 fails to stably associate with the pre-60S particle, just as we showed previously for the Drg1-18 protein^8^. As a consequence, shuttling proteins (exemplified by Nog1 and Rlp24) and export factors (Mex67 and Mtr2) accumulated on the pre-60S particles, while late joining proteins (Sqt1 and Rpl10) showed reduced levels. These findings are in line with results obtained with the Drg1-18 protein and suggest loss of function of Drg1-21. However, in contrast to *drg1-18*, which is recessive, *drg1-21* shows a dominant negative behaviour which is not easy to reconcile with a failure of the protein to bind to the pre-ribosome substrate. We therefore also purified pre-60S particles from a wildtype strain overexpressing the Drg1-21 protein. As shown in Fig. 2b, lower amounts of GST-Drg1-21 were recovered with the Arx1-TAP particles compared to overexpressed wildtype GST-Drg1 suggesting that it binds less efficiently or is lost more rapidly during purification. Nevertheless, more GST-Drg1-21 was present on the Arx1-TAP particles (lane 2) than wildtype Drg1 in the non-overexpressing strain (vector control). Moreover, the untagged wildtype copy of Drg1 was not detected in the purification of wildtype GST-Drg1 (lane 1) or GST-Drg1-21 (lane 2) overexpressing strains. This indicates that both, GST-Drg1 and GST-Drg1-21 are capable of competing with untagged wildtype Drg1 for binding. However, albeit the high levels of Drg1-21 on the Arx1-TAP particle compared to the vector control, shuttling proteins (as exemplified by Nog1) and export factors (as exemplified by Mex67) accumulated on the pre-60S particles of the Drg1-21 overexpressing strain. The accumulation of pre-60S factors suggests a defect in substrate release and shows that Drg1-21, even when it binds to the pre-60S particle, is unable to perform its function, which likely explains the dominant negative behaviour.

### The Drg1-21 variant shows a dysregulated ATPase activity

**Figure 3:**
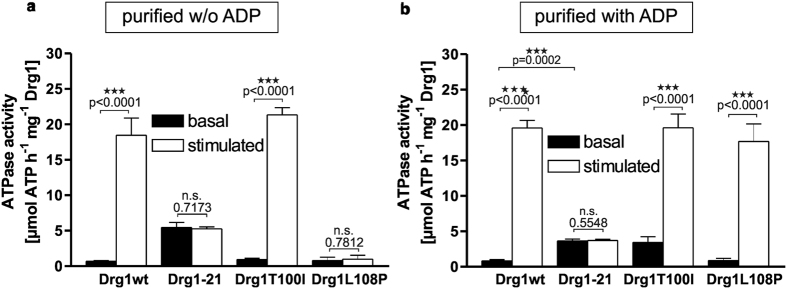
Drg1-21 shows a dysregulated ATPase activity. Drg1 wildtype as well as mutant variants were purified and the *in vitro* ATPase activity was measured using the Malachite green phosphate assay. The ATPase activity was determined in the absence (basal activity) and in the presence (stimulated activity) of the HIS_6_-tagged C-terminal fragment of the substrate protein Rlp24 (amino acids 147-199). The ATPase activity was measured for proteins purified in the absence **(a)** or presence **(b)** of 1 mM ADP. All values are presented as specific ATPase activity (μmol ATP h^−1^ mg^−1^ Drg1). Error bars represent standard deviations of means calculated from at least two biological replicates each measured in triplicate. P-values were calculated by an unpaired t-test (n.s.: not significant; p > 0.05).

Since the Drg1-21 protein, despite binding to the pre-60S particle, was unable to perform the release of shuttling proteins, we examined whether the two exchanges in the N-domain affect the ATPase activity of Drg1-21 (Fig. 3). In addition, we analysed the effects of the single exchanges. Our previous studies showed that the ATPase activity of both domains of Drg1 is stimulated by the C-terminal fragment of the substrate protein Rlp24 (Rlp24C)^8^. Therefore we also determined the stimulated ATPase activity in the presence of Rlp24C. Notably, the basal activity of the Drg1-21 variant was increased 5-fold compared to wildtype Drg1 (Fig. 3a). This elevated basal ATPase activity of Drg1-21, however, was not further stimulated by Rlp24C. Examination of the proteins containing the single exchanges demonstrated that both showed basal ATPase activity on wildtype level. While Drg1-T100I was stimulated by Rlp24C, Drg1-L108P showed no increase in ATP hydrolysis in the presence of the substrate. Interestingly, we found that the addition of ADP during the purification procedure significantly increased the yield of otherwise badly purifying Drg1-21 and Drg1-L108P, but decreased the yield of wildtype Drg1 and Drg1-T100I. This prompted us to analyse the ATPase activity of proteins purified in the presence of ADP (Fig. 3b). Under these conditions the Drg1-T100I variant exhibited increased basal activity like Drg1-21, whereas Drg1-L108P showed the same basal activity as the wildtype protein (Fig. 3b). This indicated that the T100I exchange is responsible for the elevated basal activity of Drg1-21 and that this effect is ADP dependent. Interestingly, the failure of Drg1-L108P to be stimulated by Rlp24C was not observed when the protein was purified in the presence of ADP. This is in sharp contrast to Drg1-21 that exhibited elevated basal activity and a failure to be stimulated under both purification conditions (Fig. 3a,b). Taken together these results suggest that the two exchanges in Drg1-21 exhibit a synergistic behaviour which is modulated by ADP.

### Drg1-21 fails to dissociate from the substrate protein Rlp24

**Figure 4:**
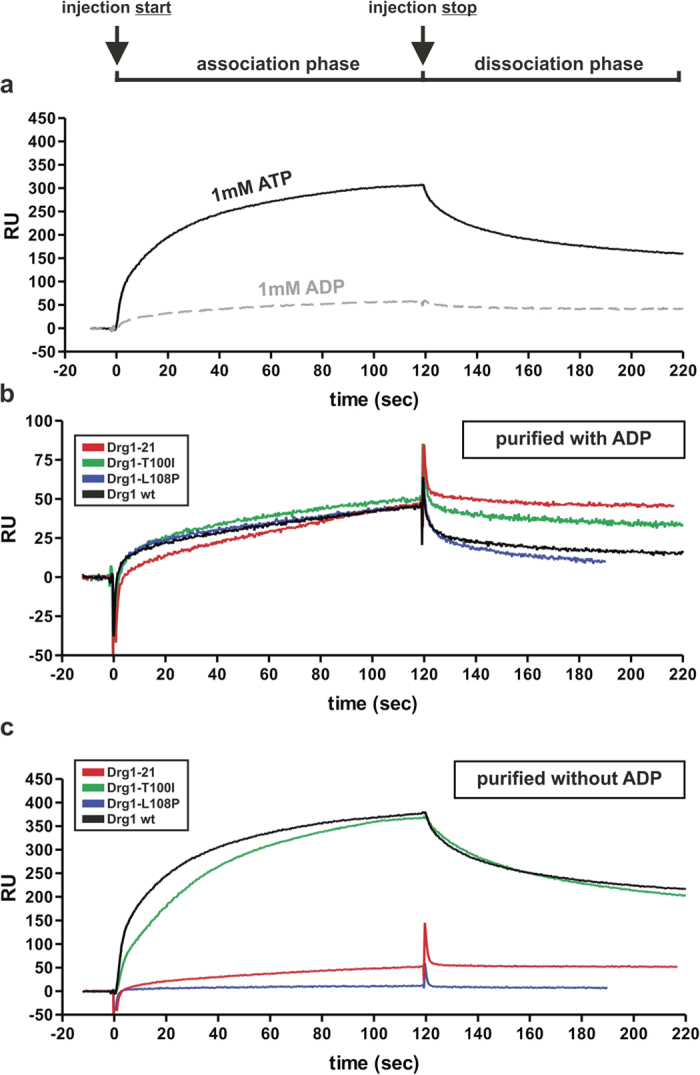
The Drg1-21 variant fails to dissociate from the substrate protein Rlp24. Surface plasmon resonance measurements were performed on a BiacoreX system. GST-tagged Rlp24C was immobilized to CM5 sensor chips (GE Healthcare) and 100 nM of the respective Drg1 variants were injected as binding partners. The reference chamber contained empty GST. The association phase starts with the injection of the sample (0 seconds) and the dissociation phase begins at the end of the injection (120 seconds). Prior to injection, the Drg1 variants were pre-incubated with 1 mM ATP for 5 minutes. (**a**) Drg1 wildtype was purified in the absence of nucleotide and pre-incubated either with 1 mM ADP or 1 mM ATP. (**b**) Drg1 variants purified in the presence of 1 mM ADP were injected. All samples were incubated with 1 mM ATP prior to injection. The running buffer contained 1 mM ATP to analyse dissociation. (**c**) Drg1 variants purified in the absence of 1 mM ADP were injected. All samples were incubated with 1 mM ATP prior to injection. The running buffer contained 1 mM ATP to analyse dissociation. The displayed data are taken from representative experiments out of at least two biological replicates collected from two CM5 chips. (RU Response units).

In order to examine whether the dysregulation of the ATPase activity of Drg1-21 is the result of an altered interaction with the substrate protein Rlp24, we performed *in vitro* binding experiments using Surface Plasmon Resonance (SPR). We immobilized GST-Rlp24C and injected purified Drg1 variants to analyse their interaction properties with the substrate. We demonstrated previously that Drg1 requires the presence of nucleotides to form hexamers and that only the hexameric form of the protein is able to interact with Rlp24C^8^. Consequently, during the dissociation phase in Surface Plasmon Resonance, Drg1 detached from Rlp24C rapidly in the absence of ATP, but dissociated more slowly when ATP was constantly present^8^. Interestingly, binding to Rlp24C was much stronger when Drg1 was pre-incubated with ATP than with ADP (Fig. 4a). Therefore, 1 mM ATP was added to the Drg1 preparations five minutes prior to injection as well as to the running buffer.

Interestingly, the wildtype protein purified in the presence of ADP showed lower response units than the protein purified in the absence of nucleotide (compare Fig. 4b,c, note the different scales). Since the protein was pre-incubated with ATP in both cases, this finding suggests that ADP was stably bound to one of the two ATPase domains and could be less efficiently displaced by ATP. The importance of nucleotide binding for the interaction with Rlp24C prompted us to investigate the contribution of the two domains. To dissect the individual sites we tested Drg1 variants containing K292A (KA1) and K563A (KA2) exchanges in the Walker A motifs of each ATPase site. These exchanges are known to prevent nucleotide binding in the respective domains, allowing selective examination of the binding properties of the non-mutated domain. As shown in Fig. S2, the Drg1-KA1 protein did not interact with Rlp24C, while Drg1-KA2 was still able to bind to Rlp24C but dissociated more rapidly from the substrate compared to wildtype. This finding suggests that ATP binding into D1 is essential for substrate recognition and loading of D2 with ATP is required to provide stable association. Measurement of ATP binding to these KA mutants using differential scanning fluorimetry (DSF) suggests a K_d(ATP)_ of about 20 μM for the D1 domain and about 80 μM for the D2 domain (Fig. S3). These values are in good agreement with earlier estimates obtained by radioactive nucleotide binding studies with wildtype Drg1^27^. With respect to ADP binding we measured a K_d(ADP)_ of about 120 μM for the D1 domain, while binding to D2 was below the detection limits of our experimental setup. Together with our previous finding that blockage of ATP hydrolysis in D1 results in more stable interaction with the substrate^8^, these results suggest that the D1 domain is the main determining factor for substrate interaction. Therefore we speculate that ADP is stably bound to D1 during purification and cannot easily be displaced by ATP added during the pre-incubation and the association phase of the SPR experiments. However, since nucleotide loading of D2 is required for stable interaction (Fig. S2), we cannot exclude that ADP binding into this domain also modulates substrate interaction.

The different behaviour of the Drg1-21, Drg1-T100I and Drg1-L108P variants in respect to stimulation of ATPase activity depending on purification in the presence or absence of ADP prompted us to investigate each protein for binding to Rlp24C under both conditions. The Drg1-L108P protein purified in the absence of nucleotide did not interact with Rlp24C, while it showed interaction when purified in the presence of ADP (compare Fig. 4b,c). The inability to interact with the substrate protein explains the failure of Drg1-L108P purified in the absence of ADP to be stimulated for ATP hydrolysis (Fig. 3a). The Rlp24C binding characteristics of Drg1-T100I were similar to those of the wildtype protein (Fig. S4). These results indicate that the L108P exchange is the major determinant for the altered interaction of Drg1-21 with Rlp24C.

Drg1-21 showed similar binding to Rlp24C under both conditions and this binding was comparable to the binding of the other Drg1 variants purified in the presence of ADP (Fig. 4b,c). However, in contrast to the other proteins, Drg1-21 failed to be released from Rlp24C during the dissociation phase. Together our findings demonstrate a synergistic effect of the T100I and L108P exchanges also with respect to substrate interaction.

### An intragenic suppressor mutation partially restores the regulation of ATPase activity of Drg1-21

**Figure 5:**
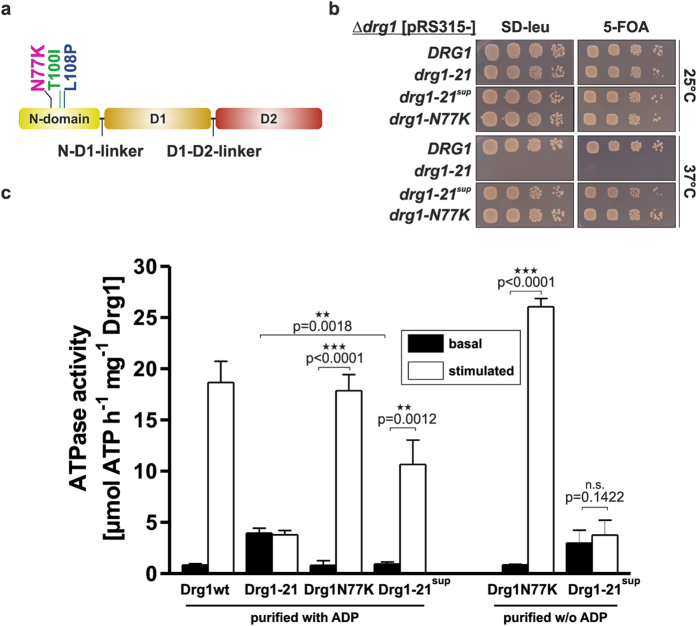
An additional N77K exchange suppresses the phenotypes of *drg1-21*. (**a**) Domain representation of Drg1 with the location of the Drg1-21 exchanges (T100I and L108P) and the additional suppressor exchange (N77K, magenta). (**b**) *Drg1* knockout strains expressing the *drg1-21*^*sup*^ allele (N77K/T100I/L108P) or the *drg1-N77K* allele (N77K) as control were spotted on SD-leu as well as on SD + 5-FOA to confirm plasmid shuffling and were incubated at 25 °C and 37 °C. (**c**) The N77K exchange partially restores the regulation of ATPase activity of the Drg1-21 variant. Drg1 wildtype as well as mutant variants were purified and the *in vitro* ATPase activity was measured using the Malachite green phosphate assay. The ATPase activity was determined in the absence (basal activity) and in the presence (stimulated activity) of the HIS_6_-tagged C-terminal fragment of the substrate protein Rlp24 (amino acids 147-199). The ATPase activity was measured for proteins purified in the presence or absence (w/o) of 1 mM ADP. All values are presented as specific ATPase activity (μmol ATP h^−1^ mg^−1^ Drg1). Error bars represent standard deviations of means calculated from at least two biological replicates each measured in triplicate. P-values were calculated by an unpaired t-test (n.s.: not significant; p > 0.05)

In order to understand the molecular mechanisms causing the failure in activation of the Drg1-21 protein by the substrate Rlp24C we screened for suppressor mutants restoring growth at 37 °C. After PCR mutagenesis of the *drg1-21* allele, we isolated 28 mutants that permitted growth at the restrictive temperature. 12 of these mutants contained reversions of at least one of the two original mutations of the *drg1-21* allele. 15 additional mutants contained codon changes leading to other amino acids instead of either I100 or P108. We identified four substitutions for lysine codons in position 100. In position 108 we found four exchanges for threonine, six for serine and one for alanine. Finally, we isolated one mutant that still contained the original exchanges (T100I and L108P) but was able to grow at 37 °C (Fig. 5a,b). This mutant carried an additional mutation leading to an N77K exchange in the N-domain and was designated *drg1-21*^*sup*^. To investigate whether the restoration of growth is due to a recovery of the stimulatability in this mutant we purified the Drg1-21^sup^ protein and analysed its basal ATPase activity and its stimulation by Rlp24C (Fig. 5c). As a control we included the Drg1-N77K variant that carries the N77K exchange alone (without the T100I/L108P exchanges). Notably, when purified in the absence of ADP, Drg1-21^sup^ could not be stimulated by Rlp24C and showed elevated basal activity, which closely reflects the situation with Drg1-21. However, both proteins, Drg1-21^sup^ and the Drg1-N77K variant, showed similar basal activity as the wildtype protein and were stimulated by Rlp24C when purified in the presence of ADP. Although Drg1-21^sup^ did not reach the full wildtype activity in the presence of Rlp24C, the regulation was partially restored.

### The defects of Drg1-21 are caused by exchanges at the N-D1 interface

**Figure 6:**
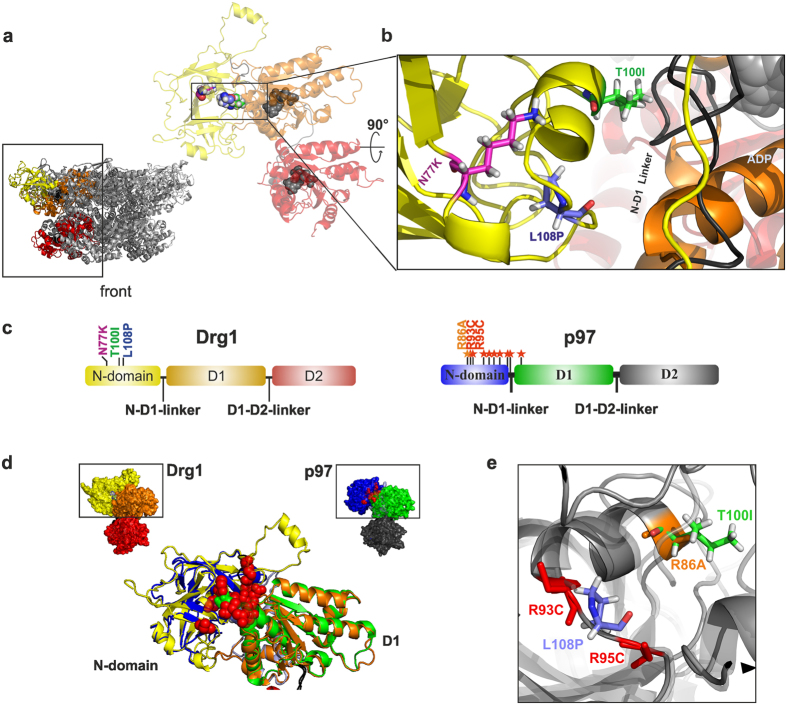
The Drg1-21 exchanges are located at the N-D1 interface similar to IBMPFD exchanges of p97. (**a**) Cartoon representation of a modelled Drg1 hexamer in front view (small image) as well as one magnified monomer highlighted in colour (N-terminal domain in yellow, N-D1 linker in black, D1 domain in orange and D2 in red; bound ADP in D1 and D2 in grey and in sphere representation). **(b)** Magnification of the N-D1 interface with the following residues shown in stick representation: the amino acid substitutions of the Drg1-21 variant (T100I in green and L108P in blue) and the suppressor exchange N77K (magenta). The ribose group of ADP bound in D1 is shown in grey. **(c)** Domain representation of Drg1 and the related mammalian AAA-ATPase p97. Drg1 residues are coloured according to (**b**). In p97, red stars mark positions of documented IBMPFD exchanges (annotated according to ref. 30). The “IBMPFD-like” exchange R86A^14^ is marked in orange. **(d)** Structural alignment of the N-D1 segments of Drg1 and p97. The exchanges present in Drg1-21 or in p97 IBMPFD variants are coloured as in (**c**). (**e**) Magnified section of the overlay of the modelled structure of Drg1 (light grey, transparent) with the structure of p97 (dark grey, solid). Highlighted residues are coloured as in (**c**).

Structural investigations of p97 showed that the protein can adopt different conformations depending on the nucleotide-binding state of the ATPase domains^7^. In the presence of ADP the N-domain is positioned coplanar with the D1 ring (down-position), while in the presence of ATPγS it is found elevated above the D1 ring (up-position)^12^. These positions are supposed to be in a dynamic, nucleotide-dependent equilibrium^26^. Structural investigations of the pathogenic IBMPFD mutants showed that they preferentially adopt the up-position even in the ADP-bound state and shift the equilibrium to the elevated position^13^,^26^. To investigate the structural impact of the Drg1-21 mutations, we analysed our previously built homology model of Drg1^29^ as well as five newly built models based on the recently released high resolution cryo-EM structures of human p97^12^. While the alignment in the D1 and D2 domains is highly confident with about 50% sequence identity (Fig. S1), the N-domains of Drg1 and p97 have diverged considerably, making homology-based conclusions more difficult. Nevertheless, we found a 23-residue stretch in the N-domain (Drg1 residues I95 to K117) where the alignment is securely anchored based on several conserved residues including the triplet ‘LGD’ and a perfect match of predicted and actual secondary structure in p97 (Fig. S1). To further validate our predictions we compared several N-domain models based on different template structures (including high resolution cryo-EM structures of p97 and more distantly related templates). Comparison of these models suggests that the general architecture of the N-domains of these proteins is well conserved (Fig. S5). Compared to p97, the N-domain of Drg1 contains an N-terminal extension of 28 residues and an insertion from residue 136 to 156 for Drg1 (Fig. S1). Nevertheless, all models based on p97 structures with the N-domain in the down-position predicted positioning of T100I and L108P at the N-D1 domain interface (Fig. S5; Fig. 6a,b).

Remarkably, the location of these residues resembles the positions of exchanges in p97 leading to the hereditary disease IBMPFD/MSP1, which also cluster at the contact area between these two domains (Fig. 6c,d). Our models predict that threonine 100 of Drg1, which is changed to isoleucine in the *drg1-21* mutant, faces towards the N-D1 linker. This linker is thought to play an important role in signal transduction from the N- to the D1 domain^14^,^19^. Moreover, in a superposition of the Drg1 model and the p97 structure, shown in Fig. 6e, T100 matches with arginine 86 of p97, which is known to cause IBMPFD-like features when mutated to alanine^14^. The exchange of leucine 108 for proline seems to be the major determinant for the temperature sensitivity and growth phenotype of *drg1-21*. In the superposition it is located between two p97 residues, arginines 93 and 95, for which IBMPFD/MSP1 exchanges were reported^30^. This further strengthens the hypothesis that the *drg1-21* mutations resemble those pathological exchanges in p97.

According to our Drg1 structural model, the N77K exchange, which suppresses the temperature sensitive growth phenotype of the *drg1-21* allele, is located at the surface-exposed side of the N-domain in the down-position. As a consequence of the structural change of the N-domain from the down- to up-position, however, N77 is relocated towards the D1 domain (Fig. S6). The insertion of the larger lysine residue in this position instead of the smaller asparagine might lead to structural changes at the N-D1 interface which are likely connected to the suppression mechanism.

## Discussion

Drg1 and p97 (or its yeast orthologue Cdc48) act in physiologically unrelated pathways. While Drg1 is specific for one distinct pre-ribosomal maturation step, p97 acts in a variety of cellular pathways by means of different adaptor proteins^7^,^31^,^32^. Nevertheless, our data suggest that both proteins use conserved mechanisms for the regulation of ATPase activity.

This idea became obvious by comparing both, structural and functional features of the IBMPFD variants of p97 on the one hand and the Drg1-21 variant on the other hand. These include positioning of the exchanges at the N-D1 interface^30^, elevated basal ATPase activity^13^,^18^,^25^, changes in adaptor protein/substrate binding^13^,^33^ and a dominant negative phenotype upon overexpression^34^. In addition, a failure to stimulate ATPase activity through binding of adaptor (p37 for p97^18^) or substrate proteins (Rlp24 for Drg1) was also found for mutated forms of both AAA-ATPases. In contrast to p97, where single exchanges cause these phenotypes, we have found a combination of two mutations to be responsible in Drg1-21. None of the single exchanges manifests the full severity of the phenotypes observed for the Drg1-21 variant but they exhibit a synergistic behaviour with respect to ATPase activity, substrate interaction and dominant negative growth phenotype. Interestingly, the behaviour of the proteins containing the single exchanges was modulated by the presence of ADP. This indicates an important role of this nucleotide for causing the deleterious effect of Drg1-21.

For some p97-IBMPFD variants it was demonstrated that they co-precipitate increased levels of ubiquitinated proteins^34^ (their native substrates) suggesting a substrate release defect. In addition, altered nucleotide-dependent binding and a strongly reduced dissociation from the adaptor proteins p37 and p47 was shown for the p97-IBMPFD variants R155H and A232E^33^. Analogously, Drg1-21 fails to be released from its substrate Rlp24. Despite all these similarities, p97 and Drg1 differ with respect to the nucleotide states of the isolated proteins, as purified p97 usually retains ADP bound to at least some of its ATPase domains^7^. For Drg1, nucleotide binding to D1 is crucial to allow initial substrate recognition, but subsequent loading of ATP into D2 stabilizes the interaction. Due to this complex relationship, we cannot determine yet, how exactly ADP interferes with substrate binding and stimulation of ATP hydrolysis. However, the differential effects of ADP on Drg1-T100I and Drg1-L108P suggest the existence of an ADP containing intermediate state which is crucial for substrate handling. Based on the higher affinity of D1 we speculate that the ADP-dependent effects are linked to ADP binding into D1. To unravel the role of ADP in Drg1 regulation, high resolution structures of Drg1 bound to different nucleotides will be required.

The dysregulation of Drg1-21 suggests that binding of the substrate cannot be transmitted to the ATPase domains and the N-domain cannot fulfil its regulatory function. According to our structural models, T100 corresponds to R86 of p97. A mutation to alanine at this position is linked to “IBMPFD-like” structural alterations in p97^14^. Disturbed interactions of the N-domain with residues of the N-D1 linker were suggested to be the molecular basis for the conformational changes of the p97-R86A variant. In all calculated structural models of Drg1, T100 is predicted in close proximity to the N-D1 linker and mutating this residue is sufficient to increase the basal ATPase activity of Drg1. This further corroborates that interactions at the N-D1 interface are crucial for the regulation of ATP hydrolysis. Importantly, the Drg1-T100I exchange alone does neither cause temperature sensitivity nor exhibit a dominant negative effect on growth. Therefore, the enhanced ATP hydrolysis of Drg1-21 is not directly linked to the dominant negative behaviour but might rather be a consequence of disturbed interactions between N-domain and D1. Only the additional L108P substitution, which itself only causes a mild temperature sensitive phenotype and a mild dominant negative growth defect, results in full appearance of the deleterious effects of Drg1-21. We conclude that the two exchanges present in the Drg1-21 variant uncouple the combination of phenotypes caused by single amino acid exchanges in p97-IBMPFD variants and could therefore help to dissect the mechanism underlying the disease. Our results raise the speculation that the elevated basal ATP hydrolysis may not be causally linked to the pathogenesis, but might merely be a consequence of the failure of the N-domain to correctly interact with substrate and adaptor proteins and transmit a regulatory signal via the N-D1 linker.

Our screen for suppressor mutants showed that incorporation of a polar sidechain at position 100 of Drg1-21 is sufficient to restore activity of the protein. Polar and hydrophobic interactions at the N-D1 interface seem to be critical determinants for the positioning of the N-domain relative to the D1 domain. In p97, R86 forms multiple polar contacts with residues of the N-D1 linker (D204 and D205) and similar interactions were also predicted for the IBMPFD residues R93 and R95^14^. These interactions are lost when the N-domain changes its position. In other mutants, identified by the suppressor screen, the rigid proline in position 108 was exchanged for more flexible residues. Proline108 (L108P) might further dislocate residues responsible for inter-domain contacts (including threonine 100) and thereby explain the synergistic effect of both mutations. Interestingly, the only suppressor that retained the two original exchanges had an additional N77K substitution. This change obviously compensates for alterations introduced by T100I and L108P and therefore allows a positioning of the N-domain relative to the D1 domain that restores its function as regulator of ATPase activity. The fact that Rlp24C is able to stimulate ATP-hydrolysis by the Drg1-21^sup^ protein explains the suppression of the temperature sensitive phenotype of Drg1-21. In cryo-EM structure of p97 obtained in the presence of ATPγS, where the N-domain adopts an elevated position reminiscent of the IBMPFD variants, residue R65 (which corresponds to N77 in Drg1), is rotated towards the N-D1 linker which undergoes a loop to helix transition upon ADP to ATPγS loading into D1^14^. Recently, NMR experiments showed that the N-D1 linker is an important component in transmitting perturbations of the N-domain “up/down” equilibrium in the IMBPFD mutant R95G to the nucleotide binding pocket of D1 (pathway I^26^). It is therefore tempting to speculate that the N77K exchange in Drg1 reverses the defects of Drg1-21 by interfering with the N-D1 linker. This could shift the up/down equilibrium of the N-domain towards the down-position and thus rescue the phenotype of the double mutation in Drg1-21. However, in the absence of an experimentally determined structure for Drg1, and given the fact that the region containing the N77 residue is less confidently represented in our Drg1 models, these explanations are largely speculative. Therefore the exact mechanism behind the suppressor exchange N77K has to remain elusive and awaits further investigations.

Our findings suggest that the primary defect in the Drg1-21 mutant is the altered interaction between N- and D1-domain which might be counteracted by conformational rearrangements at the N-D1 interface. Taken together, the characterization of the Drg1-21 variant gave new insights into the regulation of ATPase activity of Drg1 as one of the crucial trans-acting enzymes during eukaryotic ribosome biogenesis. Our findings revealed a striking correlation with the pathological IBMPFD variants of p97. This indicates that despite low sequence conservation of the N-domain, the basic mechanisms of regulation of the ATPase activity are conserved among AAA-ATPases. In this context our work also contributes to the understanding of the molecular basis of the deleterious effects caused by dysregulated AAA-ATPases, including IBMPFD variants of p97.

## Experimental procedures

### Yeast Strains and Growth Conditions

All yeast and bacterial strains used in this study are listed in Supplementary Table S1, all plasmids are listed in Supplementary Table S2. Chromosomal deletions or gene fusions were generated by homologous recombination with PCR generated fragments as previously described^35^. Strains were grown either in YPD complex medium or for plasmid maintenance in synthetic dextrose complete medium supplemented with an appropriate amino acid mix to maintain selection. SD + all amino acids supplemented with 1 g/l 5-fluoroorotic acid (5-FOA, Thermo scientific) were used for plasmid shuffle experiments. Strains carrying temperature sensitive alleles were either incubated at permissive (25 °C) or restrictive (37 °C) temperatures.

### Screening for temperature sensitive *DRG1* mutants

Temperature sensitive alleles were generated by error prone PCR mutagenesis in the presence of 0.05 mM Mn^2+^. The mutagenized PCR fragments were cloned into a centromeric vector (pRS315) and transformed into the heterozygous diploid W303 *DRG1/drg1-1::URA3* strain. After sporulation, random spore analysis was used to select for cells carrying the disrupted chromosomal copy of *DRG1* as well as the mutagenized allele on the plasmid. Temperature sensitive alleles were identified by replica plating and incubation at permissive (25 °C) and restrictive (37 °C) temperatures. Localization of the mutations was identified by sequencing.

### Construction of *DRG1* shuffle plasmids

The centromeric plasmids containing the *drg1-21* allele and the alleles with the single mutations were generated by overlap-PCR using primer pairs carrying point mutations followed by intragenic fragment swapping to exchange fragments from pRS315-*DRG1* for mutagenized PCR fragments^36^. Mutagenic primers are listed in Supplementary Table S3.

### Expression and purification of Drg1 variants

The pCUP1 expression plasmids in this study were derived from the pAZ7 (*GST-DRG1*) plasmid described in ref. 27. These constructs contain a uracil auxotrophy marker, the copper-inducible *CUP1*-promoter and an N-terminal GST-tag, which is separated from the *DRG1*-alleles by a PreScission^TM^ protease (GE Healthcare) site to allow removal of the GST-tag. The mutant *drg1* alleles were introduced by intragenic fragment-swapping. The expression protocol of the Drg1 variants in this study is a modified version of the protocol for wildtype Drg1 which is described in detail elsewhere^8^,^29^. For expression of the Drg1-21 variant and the single mutant variants (L108P and T100I) two to twelve litres of SD-ura media were inoculated to an OD_600_ of 0.01 in baffled flasks and incubated at 25 °C under constant shaking at 110 rpm. At an OD_600_ of 0.2, protein expression from the *CUP1* promoter was induced by adding 0.1 mM CuSO_4_. After another 18 hours at 25 °C the cells were harvested by centrifugation, washed with aqua bidest. and stored at −80 °C. Purification of the GST-fusion proteins was performed essentially as described in ref. 8 except that the pH of the crude extracts was adjusted to 7.4 with Tris base. When indicated 1 mM ADP was present in all purification buffers, since ADP proved to have a positive effect on the yield of the otherwise poorly purifying mutant fusion proteins. The concentration of the eluted proteins was determined using the Bradford assay (Biorad).

### Tandem affinity purification of pre-60S particles

Tandem affinity purifications of pre-60S particles were performed as described in ref. 8 adapted from the standard protocols^37^,^38^. Cells were grown in YPD to an OD_600_ of 2.0, harvested by centrifugation and stored at −80 °C. For overexpression of the *DRG1* alleles from pCUP1-plasmids, the cells were grown in SD-ura to an OD_600_ of 1.0 (early log-phase) and overexpression of the GST-Drg1 variants was induced by addition of 0.5 mM CuSO_4_ for 3 hours. Afterwards, cells were harvested by centrifugation and stored at −80 °C. Cell lysis and pre-60S particle purification was performed as described in ref. 8. After elution from calmodulin beads, proteins were concentrated by TCA precipitation.

### SDS-PAGE and Western Blotting

Protein samples were separated using NuPAGE™ Novex™ 4–12% Bis-Tris gradient Protein Gels (Thermo scientific). Western blot detection of specific proteins after transfer to PVDF membranes (Millipore) was described in ref. 8 including sources of the used antisera.

### Affinity purification of Rlp24C

Purification of the Hexahistidine-tagged (HIS_6_) and GST-tagged Rlp24C fragments was performed as described in detail in ref. 8.

### ATPase activity assay (Malachite green phosphate assay)

ATPase activity was measured using the Malachite green phosphate assay (Bioassay systems) and was carried out as described previously^8^,^29^ with the exception that 5 μg (98 nM hexamer) of Drg1 or its variants were used. The absorbance of the samples at 600 nm was measured using a TECAN^TM^ plate reader (GeniusPro). For the measurement of the stimulated activity, saturating concentrations of purified HIS_6_-Rlp24C (1.68 μM) were added. All values were calculated as specific activity (μmol ATP h^−1^ mg^−1^ Drg1. Error bars represent standard deviations of means calculated from at least two biological replicates each measured in triplicate. P-values were calculated by an unpaired t-test (two-tailed, 95% confidence intervals); for pairs with unequal variances (confirmed by F-test) p-values were calculated by an unpaired t-test with Welsh’s correction. All calculations were performed using the GraphPad^TM^ Prism software (version 3.03).

### Surface plasmon resonance

SPR binding experiments with immobilized GST-Rlp24C were performed on a BiacoreX^TM^ system as described previously^8^. Purified Drg1 variants were injected at concentrations of 10-100 nM at a constant flow rate of 40 μl/min (120 seconds contact time) at a constant temperature of 22 °C. As in-line control the signal measured in the control flow cell (immobilized GST-tag) was subtracted for each sensorgram. After each cycle, the binding surface was regenerated using running buffer containing 1 M sodium chloride to remove bound analyte. Sensorgrams were analysed using the BIAevaluation software (version 3.2). Kinetic parameters were calculated using the BIAevaluation software (version 3.2) and fitting to a two state (conformational change) model. Data were collected from at least two biological replicates over at least two CM5 sensor chips

### Suppressor screening

Generation of random mutations of the *drg1-21* allele was performed by mutagenic PCR in the presence of 0.05 mM Mn^2+^. The mutagenized PCR fragments were cloned into centromeric pRS315-plasmids and used for transformation of the *drg1-21* shuffle strain [pRS316-*drg1-21*]. To identify mutations that suppressed the temperature sensitivity caused by the *drg1-21* allele, the transformants were plated on SD-leu agar plates and incubated at 37 °C. Plasmid shuffling was performed on SD-agar plates containing 1 g/L 5-FOA (5-Fluorootic acid). Plasmids of transformants with restored growth at 37 °C were isolated and the location of the mutation was identified by sequencing.

### Generation of a Drg1 homology model based on p97 crystal structures

For structural analysis of the mutants, we re-used the model built previously with YASARA^29^,^39^ based on murine p97 crystal structures 3CF3 and 1R7R as templates, and with the same protocol built five more models using the recently released cryo-EM structures of human p97^12^ (PDB codes 5FTJ, 5FTK, 5FTL, 5FTM and 5FTN). Modelling was also performed using the Phyre^2^ web service for fold recognition^40^. The Images were generated with PyMOL (The PyMOL Molecular Graphics System, Version 1.3.0.0 Schrödinger, LLC) which was also used for structure alignment. PDB files of all models are available from the authors upon request.

## Additional Information

**How to cite this article:** Prattes, M. *et al*. A conserved inter-domain communication mechanism regulates the ATPase activity of the AAA-protein Drg1. *Sci. Rep.* **7**, 44751; doi: 10.1038/srep44751 (2017).

**Publisher's note:** Springer Nature remains neutral with regard to jurisdictional claims in published maps and institutional affiliations.

## Supplementary Material

Supplementary Information

## References

[b1] Hanson P. I. & Whiteheart S. W. AAA+ proteins: have engine, will work. Nat. Rev. Mol. Cell Biol. 6, 519–529 (2005). 16072036 10.1038/nrm1684

[b2] Snider J., Thibault G. & Houry W. A. The AAA+ superfamily of functionally diverse proteins. Genome Biol. 9, 216 (2008). 18466635 10.1186/gb-2008-9-4-216 PMC2643927

[b3] Vale R. D. Aaa Proteins. J. Cell Biol. 150, f13–f20 (2000). 10893253 10.1083/jcb.150.1.f13 PMC2185557

[b4] Noi K. . High-Speed Atomic Force Microscopic Observation of ATP-Dependent Rotation of the AAA+ Chaperone p97. Structure 21, 1992–2002 (2013). 24055316 10.1016/j.str.2013.08.017

[b5] Yeung H. O. . Inter-ring rotations of AAA ATPase p97 revealed by electron cryomicroscopy. Open Biol. 4, 130142–130142 (2014). 24598262 10.1098/rsob.130142 PMC3971404

[b6] Pye V. E. . Going through the motions: The ATPase cycle of p97. J. Struct. Biol. 156, 12–28 (2006). 16621604 10.1016/j.jsb.2006.03.003

[b7] Xia D., Tang W. K. & Ye Y. Structure and function of the AAA+ ATPase p97/Cdc48p. Gene 583, 64–77 (2016). 26945625 10.1016/j.gene.2016.02.042 PMC4821690

[b8] Kappel L. . Rlp24 activates the AAA-ATPase Drg1 to initiate cytoplasmic pre-60S maturation. J. Cell Biol. 199, 771–782 (2012). 23185031 10.1083/jcb.201205021 PMC3514788

[b9] Briggs L. C. . Analysis of Nucleotide Binding to P97 Reveals the Properties of a Tandem AAA Hexameric ATPase. J. Biol. Chem. 283, 13745–13752 (2008). 18332143 10.1074/jbc.M709632200 PMC2376215

[b10] Buchberger A., Schindelin H. & Hänzelmann P. Control of p97 function by cofactor binding. FEBS Lett. 589, 2578–2589 (2015). 26320413 10.1016/j.febslet.2015.08.028

[b11] Niwa H. . The role of the N-domain in the ATPase activity of the mammalian AAA ATPase p97/VCP. J. Biol. Chem. 287, 8561–8570 (2012). 22270372 10.1074/jbc.M111.302778 PMC3318706

[b12] Banerjee S. . 2.3 Å resolution cryo-EM structure of human p97 and mechanism of allosteric inhibition. Science 351, 871–875 (2016). 26822609 10.1126/science.aad7974 PMC6946184

[b13] Tang W. K. & Xia D. Altered Intersubunit Communication Is the Molecular Basis for Functional Defects of Pathogenic p97 Mutants. J. Biol. Chem. 288, 36624–36635 (2013). 24196964 10.1074/jbc.M113.488924 PMC3868774

[b14] Tang W. K. . A novel ATP-dependent conformation in p97 N–D1 fragment revealed by crystal structures of disease-related mutants. EMBO J. 29, 2217–2229 (2010). 20512113 10.1038/emboj.2010.104 PMC2905243

[b15] Huyton T. . The crystal structure of murine p97/VCP at 3.6 Å. J. Struct. Biol. 144, 337–348 (2003). 14643202 10.1016/j.jsb.2003.10.007

[b16] Tang W. K. & Xia D. Structural and functional deviations in disease-associated p97 mutants. J. Struct. Biol. 179, 83–92 (2012). 22579784 10.1016/j.jsb.2012.04.024 PMC4788498

[b17] Dreveny I. . p97 and close encounters of every kind: a brief review. Biochem. Soc. Trans. 32, 715–720 (2004). 15493996 10.1042/BST0320715

[b18] Zhang X. . Altered cofactor regulation with disease-associated p97/VCP mutations. Proc. Natl. Acad. Sci. USA 112, E1705–1714 (2015). 25775548 10.1073/pnas.1418820112 PMC4394316

[b19] Tang W. K. & Xia D. Role of the D1-D2 Linker of Human VCP/p97 in the Asymmetry and ATPase Activity of the D1-domain. Sci. Rep. 6, 20037 (2016). 26818443 10.1038/srep20037 PMC4730245

[b20] Kimonis V. E. . Clinical and molecular studies in a unique family with autosomal dominant limb-girdle muscular dystrophy and Paget disease of bone. Genet. Med. 2, 232–241 (2000). 11252708 10.1097/00125817-200007000-00006 PMC6173187

[b21] Kovach M. J. . Clinical Delineation and Localization to Chromosome 9p13.3–p12 of a Unique Dominant Disorder in Four Families: Hereditary Inclusion Body Myopathy, Paget Disease of Bone, and Frontotemporal Dementia. Mol. Genet. Metab. 74, 458–475 (2001). 11749051 10.1006/mgme.2001.3256 PMC6277059

[b22] Watts G. D. J. . Inclusion body myopathy associated with Paget disease of bone and frontotemporal dementia is caused by mutant valosin-containing protein. Nat. Genet. 36, 377–381 (2004). 15034582 10.1038/ng1332

[b23] Taylor J. P. Multisystem proteinopathy Intersecting genetics in muscle, bone, and brain degeneration. Neurology 85, 658–660 (2015). 26208960 10.1212/WNL.0000000000001862

[b24] Halawani D. . Hereditary Inclusion Body Myopathy-Linked p97/VCP Mutations in the NH2 Domain and the D1 Ring Modulate p97/VCP ATPase Activity and D2 Ring Conformation. Mol. Cell. Biol. 29, 4484–4494 (2009). 19506019 10.1128/MCB.00252-09 PMC2725746

[b25] Mountassif D., Fabre L., Zaid Y., Halawani D. & Rouiller I. Cryo-EM of the pathogenic VCP variant R155P reveals long-range conformational changes in the D2 ATPase ring. Biochem. Biophys. Res. Commun. 2015 Dec 254684636-41(2015). 10.1016/j.bbrc.2015.11.003 26549226

[b26] Schuetz A. K. & Kay L. E. A Dynamic molecular basis for malfunction in disease mutants of p97/VCP. eLife 5, e20143 (2016). 27828775 10.7554/eLife.20143 PMC5102582

[b27] Zakalskiy A. . Structural and enzymatic properties of the AAA protein Drg1p from Saccharomyces cerevisiae. Decoupling of intracellular function from ATPase activity and hexamerization. J. Biol. Chem. 277, 26788–26795 (2002). 12006565 10.1074/jbc.M201515200

[b28] Pertschy B. . Cytoplasmic recycling of 60S preribosomal factors depends on the AAA protein Drg1. Mol. Cell. Biol. 27, 6581–6592 (2007). 17646390 10.1128/MCB.00668-07 PMC2099225

[b29] Loibl M. . The drug diazaborine blocks ribosome biogenesis by inhibiting the AAA-ATPase Drg1. J. Biol. Chem. 289, 3913–3922 (2014). 24371142 10.1074/jbc.M113.536110 PMC3924260

[b30] Nalbandian A. . The multiple faces of valosin-containing protein-associated diseases: inclusion body myopathy with Paget’s disease of bone, frontotemporal dementia, and amyotrophic lateral sclerosis. J. Mol. Neurosci. MN 45, 522–531 (2011). 21892620 10.1007/s12031-011-9627-y

[b31] Franz A., Ackermann L. & Hoppe T. Ring of Change: CDC48/p97 Drives Protein Dynamics at Chromatin. Front. Genet. 7 (2016). 10.3389/fgene.2016.00073 PMC4853748 27200082

[b32] Meyer H., Bug M. & Bremer S. Emerging functions of the VCP/p97 AAA-ATPase in the ubiquitin system. Nat. Cell Biol. 14, 117–123 (2012). 22298039 10.1038/ncb2407

[b33] Bulfer S. L., Chou T.-F. & Arkin M. R. p97 Disease Mutations Modulate Nucleotide-Induced Conformation to Alter Protein-Protein Interactions. ACS Chem. Biol.(2016). 10.1021/acschembio.6b00350 PMC5224236 27267671

[b34] Erzurumlu Y. . A unique IBMPFD-related P97/VCP mutation with differential binding pattern and subcellular localization. Int. J. Biochem. Cell Biol. 45, 773–782 (2013). 23333620 10.1016/j.biocel.2013.01.006

[b35] Longtine M. S. . Additional modules for versatile and economical PCR-based gene deletion and modification in Saccharomyces cerevisiae. Yeast 14, 953–961 (1998). 9717241 10.1002/(SICI)1097-0061(199807)14:10<953::AID-YEA293>3.0.CO;2-U

[b36] Higuchi R., Krummel B. & Saiki R. K. A general method of *in vitro* preparation and specific mutagenesis of DNA fragments: study of protein and DNA interactions. Nucleic Acids Res. 16, 7351–7367 (1988). 3045756 10.1093/nar/16.15.7351 PMC338413

[b37] Rigaut G. . A generic protein purification method for protein complex characterization and proteome exploration. Nat. Biotechnol. 17, 1030–1032 (1999). 10504710 10.1038/13732

[b38] Puig O. . The Tandem Affinity Purification (TAP) Method: A General Procedure of Protein Complex Purification. Methods 24, 218–229 (2001). 11403571 10.1006/meth.2001.1183

[b39] Krieger E. . Improving physical realism, stereochemistry, and side-chain accuracy in homology modeling: Four approaches that performed well in CASP8. Proteins 77 Suppl 9, 114–122 (2009). 19768677 10.1002/prot.22570 PMC2922016

[b40] Kelley L. A., Mezulis S., Yates C. M., Wass M. N. & Sternberg M. J. E. The Phyre2 web portal for protein modeling, prediction and analysis. Nat Protoc. 10, 845–858 (2015). 25950237 10.1038/nprot.2015.053 PMC5298202

