# Supplementary material for: A conserved inter-domain communication mechanism regulates the ATPase activity of the AAA-protein Drg1
Source: Sci Rep. 2017 Mar 17;7:44751. doi: 10.1038/srep44751 (PMC5356007; doi:10.1038/srep44751)
Supplement: Supplementary Information [file srep44751-s1.pdf]

A conserved inter-domain communication mechanism regulates the ATPase activity of the AAA-protein Drg1

Michael Prattes[1], Mathias Loibl[1], Gertrude Zisser[1], Daniel Luschnig[1,2], Lisa Kappel[1,3], Ingrid Rössler[1], Manuela Grassegger[1], Altijana Hromic[1], Elmar Krieger[4], Karl Gruber[1], Brigitte Pertschy[1] and Helmut Bergler[1*]

[1]Institute of Molecular Biosciences, University of Graz, A-8010 Graz, Austria

[2]Current affiliation: Austrian Centre of Industrial Biotechnology GmbH (ACIB), Petersgasse 14, A-8010 Graz, Austria

[3]Current affiliation: Institute of Microbiology, University of Innsbruck, Technikerstrasse 25d, A-6020 Innsbruck, Austria

[4]Current affiliation: CMBI 260, NCMLS, Radboud University Nijmegen Medical Centre P. O. Box 9101, 6500HB Nijmegen, The Netherlands

*Corresponding author; Institute of Molecular Biosciences, University of Graz, Humboldtstrasse 50/EG, A-8010 Graz, Austria. Tel.: +43316380-5629; Fax: +43316380-898; e-mail: helmut.bergler@uni-graz.at.

Supplementary information

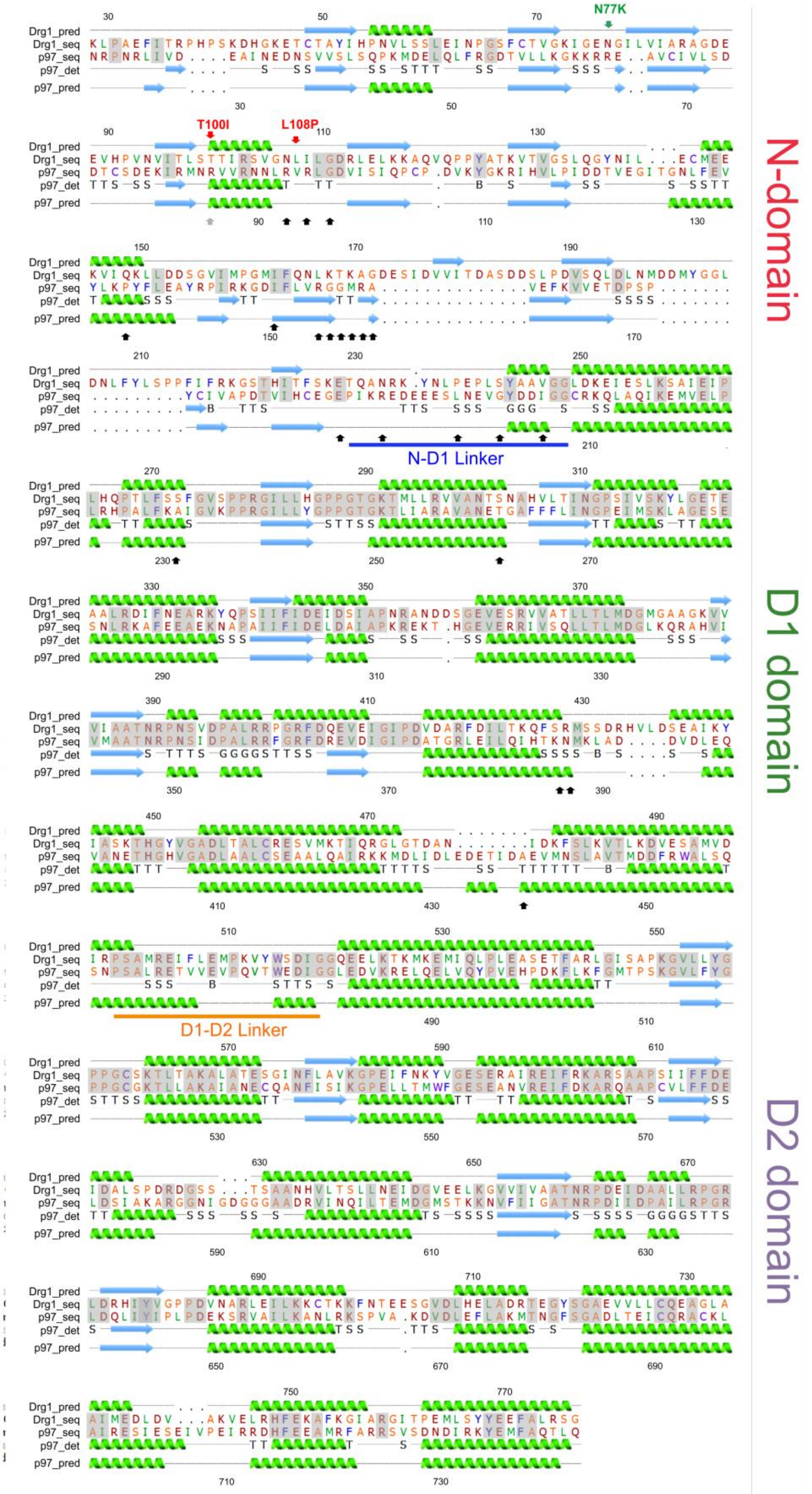

N77K
T100I
L108P
N-D1 Linker
D1-D2 Linker
Drg1_pred
Drg1_seq
p97_seq
p97_det
p97_pred
N-domain
D1 domain
D2 domain

Supplementary Figure S1: An amino acid sequence alignment of Drg1 and p97 including secondary structure prediction was generated using the web-based Phyre[2] fold recognition tool which was also used to calculate homology models[1]. The Drg1 sequence was retrieved from the SGD database (SGD ID: S000004389), human p97 was used as template (pdb code: 5FTJ). Grey highlights mark sequence identity. The secondary structure predictions of Drg1 (Drg1_pred) and p97 (p97_pred) are shown with β-strands and α-helices indicated in blue and green, respectively. For comparison, the experimentally determined structural elements for p97 (p97_det) based on pdb structure 5FTJ are shown. The p97 domain architecture was annotated according to[2]. The T100I and L108P substitutions found in Drg1-21 and the N77K additionally present in the Drg1^{sup} protein are indicated. Frequently found p97 IBMPFD exchanges are indicated by black arrows. R86A which shows IBMPFD-like properties is indicated by a grey arrowhead. In the p97_det lines, S (bend), T (hydrogen bonded turn), G (3-turn helix) and B (residue in isolated beta-bridge) refer to structural elements according to the DSSP classification[3].

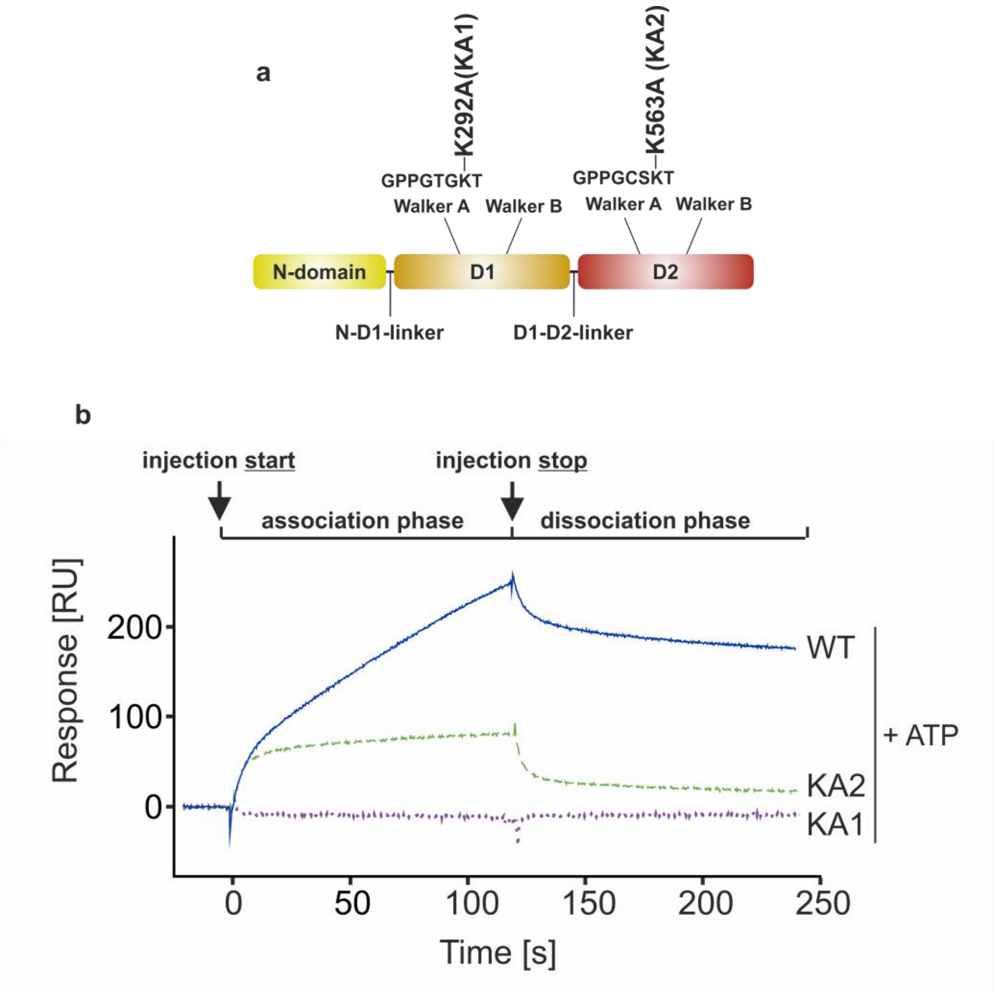

Supplementary Figure S2: (a) Location of KA exchanges in the Walker A motifs preventing nucleotide binding to the respective domains. **(b)** SPR measurements were performed to analyze Rlp24C binding of Drg1-KA mutants that show no nucleotide binding in either the D1 domain (KA1) or the D2 domain (KA2). 50 nM of the respective Drg1 variants were incubated with 1 mM ATP prior to injection at a constant flow rate of 40 µl/min (120 seconds contact time) at a constant temperature of 22°C. As in-line control the signal measured in the control flow cell (immobilized GST-tag) was subtracted for each sensorgram. The running buffer contained 1 mM ATP.

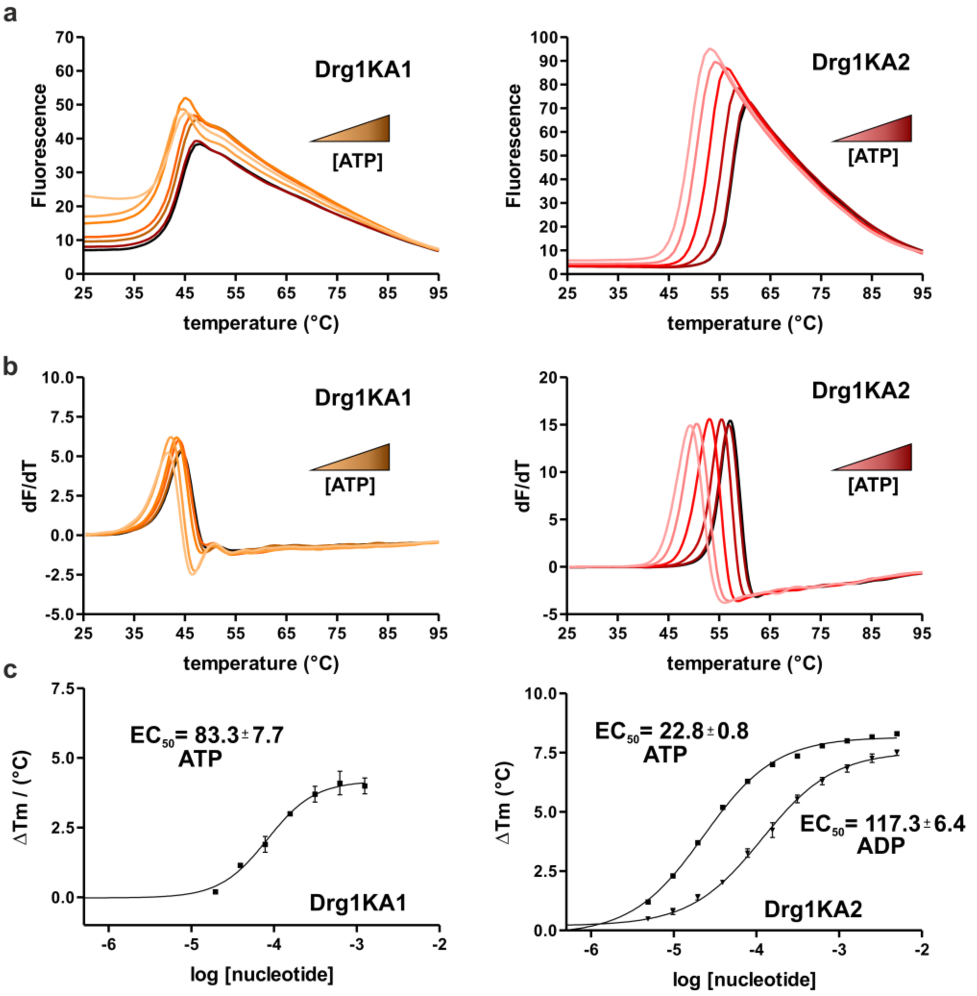

Supplementary figure S3: Nucleotide binding affinities of the Drg1-KA variants measured with Differential Scanning Fluorimetry. The experiments were performed essentially as described[4]. 10µg of the respective Drg1 variants (KA1 and KA2) were incubated with increasing concentrations of ADP or ATP (0-5 mM). **(a)** The proteins were incubated in a Corbett Rotorgene real time thermocycler with a temperature gradient from 25-95°C and thermal unfolding was monitored using the dye Sypro orange^{TM}. **(b)** Melting points (Tm) were calculated from the first derivative which represents the change of fluorescence over time (dF/dT) of the melting curves. **(c)** The change of the melting temperature (ΔTm) upon nucleotide binding was plotted against the nucleotide concentration in a logarithmic scale to calculate EC_{50} values (half maximal effective concentration). The data were fitted to a sigmoidal dose-response model. Values were calculated as means with standard deviation from two biological replicates each measured in duplicate.

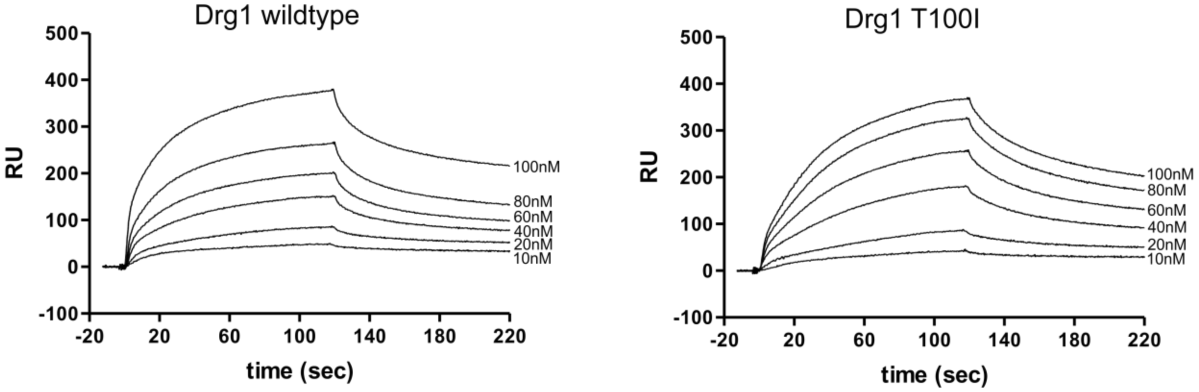

	$K_{on}1$	$K_{off}1$	$K_{on}2$	$K_{off}2$
Drg1 wildtype	4,19e+05	3,45e-02	8,32e-03	1,90e-05
Drg1T100I	1,94e+05	1,82e-02	5,61e-03	1,31e-06

Supplementary figure S4: Kinetic constants for Drg1 and Drg1T100I in the presence of ATP were calculated from SPR measurements essentially as described[5]. Proteins were purified in the absence of ADP and pre-incubated with 1 mM ATP which was also present in the running buffer at a constant concentration of 1 mM. Increasing concentrations of the proteins were injected and constants were calculated from six independent injections and fitted to a two state (conformational change) model described in[5].

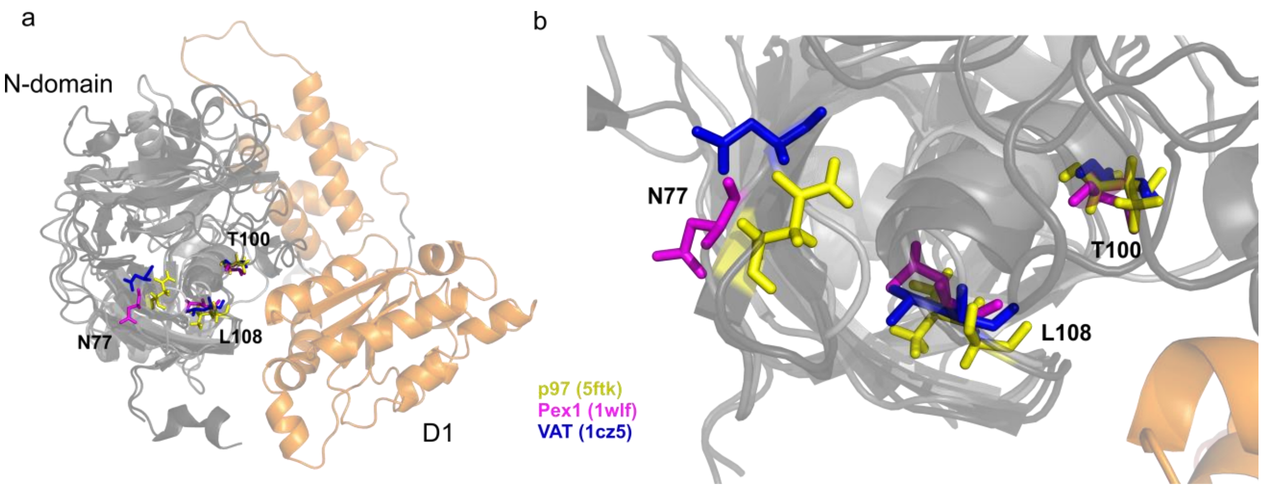

Supplementary figure S5: (a) Global superposition of modelled Drg1 N-domains based on template structures of the following AAA-ATPases (pdb code): p97 (5ftk), VAT (1cz5) and PEX1 (1wlf). The complete N-domains are shown in grey cartoon representations with predicted positions of the residues N77, T100 and L108 (magnified in **b**) highlighted according to the template structure (p97: yellow; PEX1: magenta; VAT: blue). N-domain homology models were calculated and aligned using the web-based Phyre^2 fold recognition algorithm[1]**.**

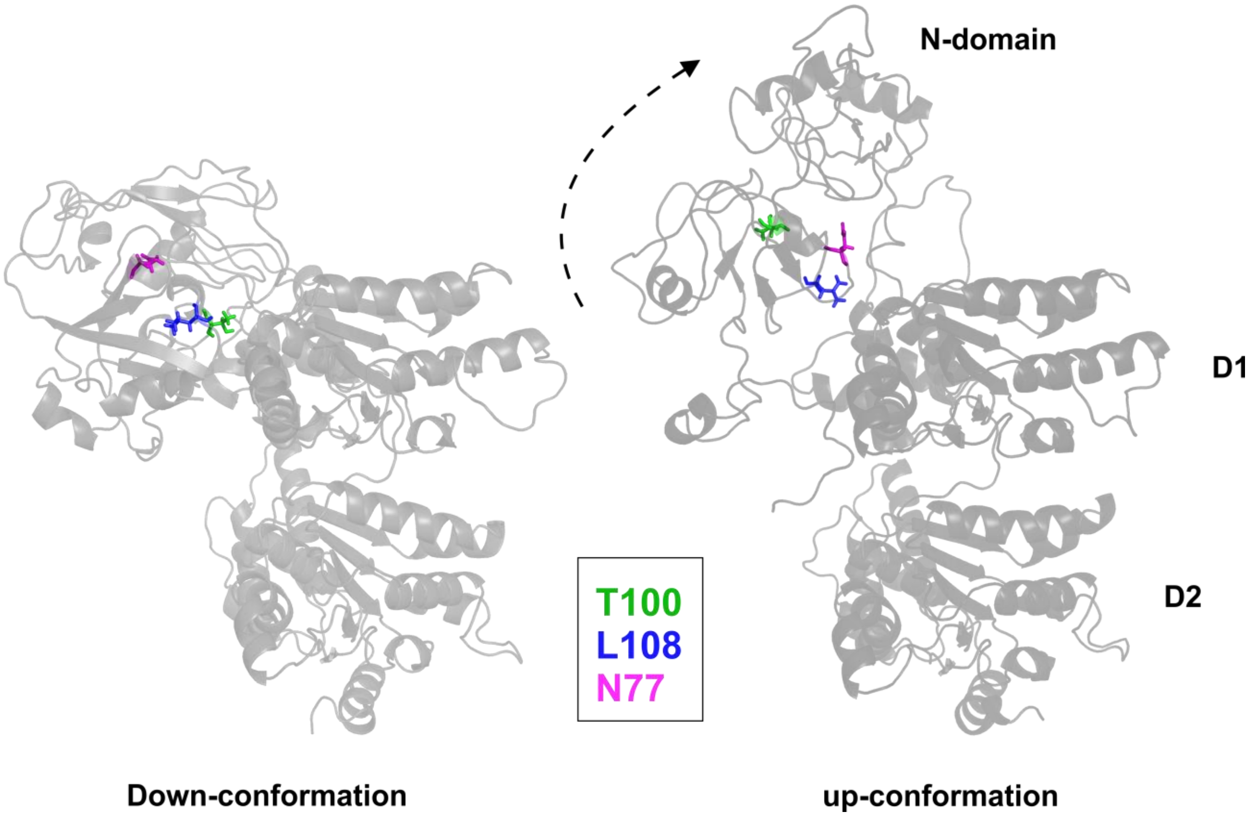

Supplementary figure S6: Modelled N-domain switch of Drg1. The structure of one Drg1 monomer was modelled based on two p97 structures in different nucleotide dependent conformations: Down-conformation (ADP, pdb code: 5ftk) and Up-conformation (ATPγS, pdb code: 5ftn). Relevant residues are depicted in color.

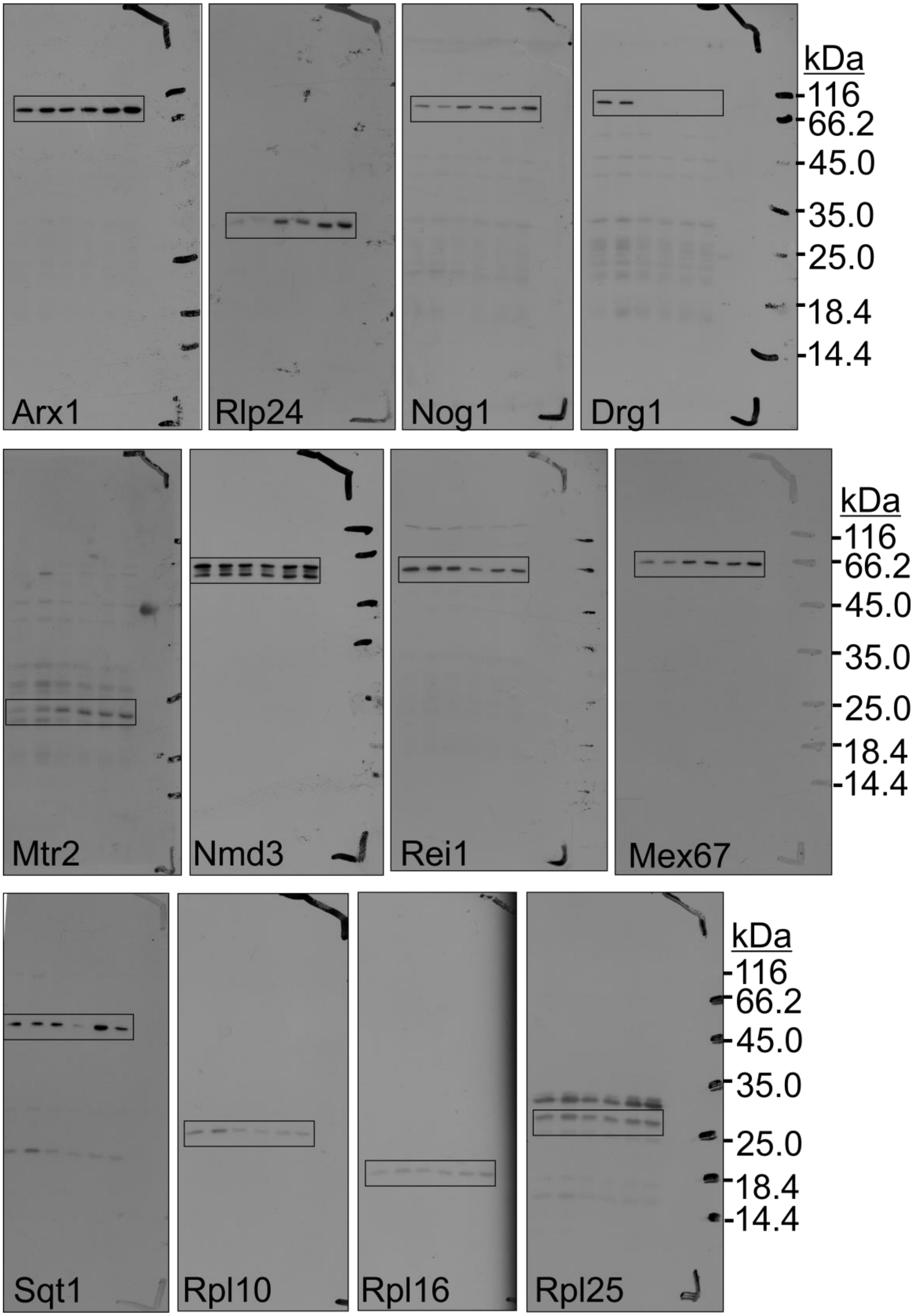

Supplementary figure S7: Full-length blots of cropped images from main Fig 2a are shown. Selections displayed in the main figure 2a are highlighted as black rectangles.

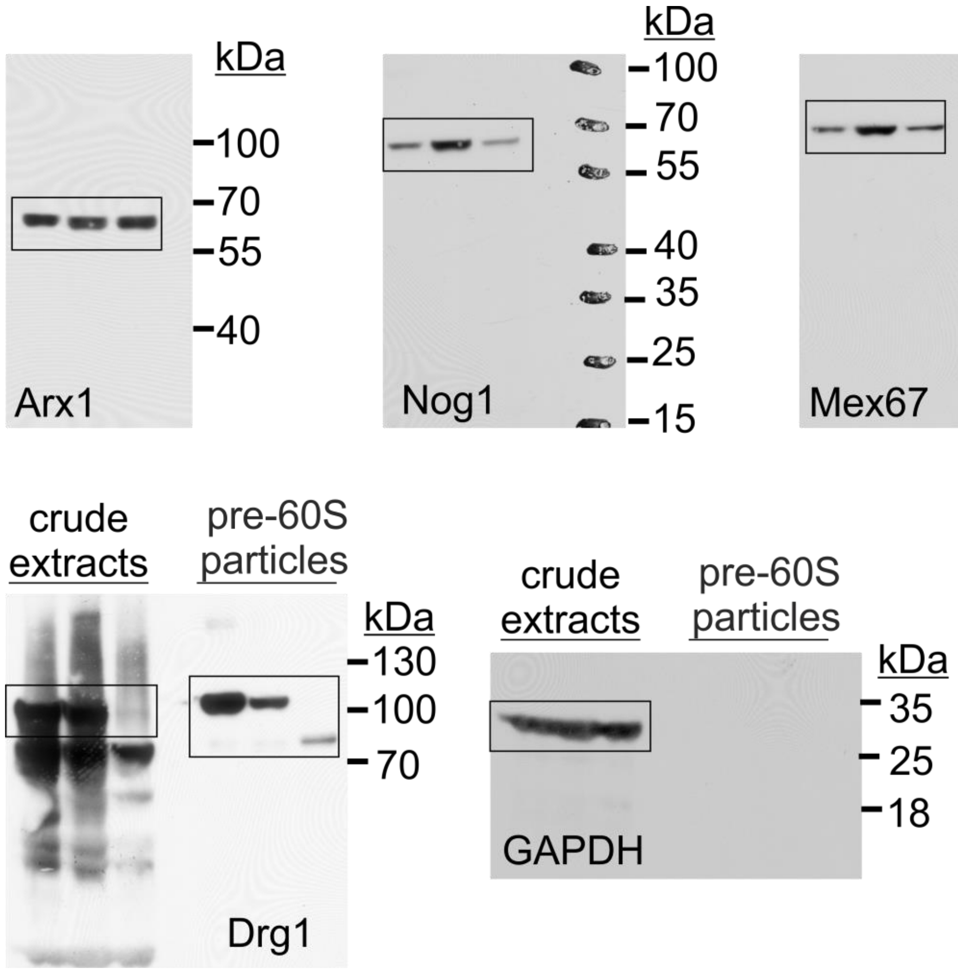

Supplementary figure S7: Full-length blots of cropped images from main Fig 2b are shown. Selections displayed in the main figure 2b are highlighted as black rectangles.

Supplementary Table S1: Bacterial and yeast strains used in this study

**Strain name**	**genotype**	**Source, experiment**
Escherichia coli		
BL21 Codon Plus (DE3)-RIPL	B F$^-$ *ompT hsdS* (rB$^-$ mB$^-$) *dcm*$^+$ Tet^r *gal λ* (DE3) *endA* Hte [*argU ileY leuW proL Cam*^{r}]	Stratagene, Expression of Rlp24C
Saccharomyces cerevisiae		
W303 *DRG1/drg1-1::URA3*	*MATa/MATα ade2/ade2 trp1/trp1 leu2/leu2 ura3/ura3 DRG1/drg1-1::URA3*	This study, Screening for *drg1-ts* mutants
BY4743 Δ*drg1/DRG1*	*MATa*/α *his3Δ1/his3Δ1 leu2Δ0/leu2Δ0 LYS2/lys2Δ0 met15Δ0/MET15 ura3Δ0/ura3Δ0 YLR397c::kanMX4/YLR397c*	Euroscarf, Expression strain for Drg1 variants
Arx1-TAP *DRG1*	*MATa ura3 leu2 his3 trp1 ARX1-TAP::TRP1 DRG1*	[6], TAP-purification of Arx1-particles/Spot assays for overexpression
Arx1-TAP *drg1-21*	*MATa ura3 leu2 his3 trp1 ARX1-TAP::TRP1 drg1-21*	This study, TAP-purification of Arx1-particles
Arx1-TAP *drg1-18*	*MATa ura3 leu2 his3 trp1 ARX1-TAP::TRP1 drg1-18*	This study, TAP-purification of Arx1-particles
DRG1 shuffle (GZAFG2)	*MATa ura3 leu2 his3 lys2 trp1 afg2::kanMX4* [pRS316-DRG1]	[4], spot assays
drg1-21 shuffle	*MATa ura3 leu2 his3 lys2 trp1 afg2::kanMX4* [pRS316-*drg1-21*]	This study, suppressor screening

Supplementary Table S2: Plasmids used in this study

Plasmid name	relevant features	Source
pCUP1-DRG1 (pAZ7)	*URA3, AmpR, ColE1, CUP1*, GST-tag, Prescission protease site, *DRG1*	[7]
pCUP1-drg1-21	*URA3, AmpR, ColE1, CUP1*, GST-tag, Prescission protease site, *drg1-21 (T100I/L108P)*	This study
pCUP1-drg1-L1008P	*URA3, AmpR, ColE1, CUP1*, GST-tag, Prescission protease site, *drg1-L108P*	This study
pCUP1-drg1-T100I	*URA3, AmpR, ColE1, CUP1*, GST-tag, Prescission protease site, *drg1-T100I*	This study
pCUP1-drg1-21^{sup}	*URA3, AmpR, ColE1, CUP1*, GST-tag, Prescission protease site, *drg1-*21^{sup} (T100I/L108P/N77K)	This study
pCUP1-drg1-N77K	*URA3, AmpR, ColE1, CUP1*, GST-tag, Prescission protease site, *drg1-N77K*	This study
pRS315-DRG1	*LEU2, AmpR, ColE1*, wildtype *DRG1* with endogenous promoter	This study
pRS315-drg1-21	*LEU2, AmpR, ColE1*, *drg1-21* (T100I/L108P) with endogenous promoter	This study
pRS315-drg1-L108P	*LEU2, AmpR, ColE1*, *drg1-L108P* with endogenous promoter	This study
pRS315-drg1-T100I	*LEU2, AmpR, ColE1*, *drg1-T100I* with endogenous promoter	This study
pRS315-drg1-21^{sup}	*LEU2, AmpR, ColE1*, *drg121*^{sup}-(T100I/L108P/N77K) with endogenous promoter	This study
pRS315-drg1-N77K	*LEU2, AmpR, ColE1*, *drg1-N77K* with endogenous promoter	This study
pRS316-DRG1	*LEU2, AmpR*, *ColE1*, *DRG1* with endogenous promoter	This study
pET32a-RLP24C	*AmpR*, N-terminal tags (TrX-tag, HIS_6-tag, S-tag), *RLP24C* (base pairs 441–599, amino acids 147-199)	[5]
pGEX-RLP24C	*AmpR*, N-terminal GST-tag, *RLP24C* (base pairs 441–599, amino acids 147-199)	[5]

Supplementary Table S3: Primers used in this study

Primer name	Sequence (5`- 3`)	Resulting mutation (codon change)
drg1-T100I_fwd	GTTATCACCCTTTCCATAACTATACGATCTGTT	C299T (ACA → ATA)
drg1-T100I_rev	AACAGATCGTATAGTTATGGAAAGGGTGATAAC	
drg1-L108P_fwd	CGATCTGTTGGGAACCCTATCCTTGGTGATCGT	T324C (CTT → CCT)
drg1-TL108P_rev	ACGATCACCAAGGATAGGGTTCCCAACAGATCG	
drg1-N77K_fwd	GTAAGATAGGCGAAAAAGGTATTTTAGTAAT	T231A (AAT → AAA)
drg1-N77K_rev	ATTACTAAAATACCTTTTTCGCCTATCTTAC	

Supplementary references

1. Kelley, L. A., Mezulis, S., Yates, C. M., Wass, M. N. & Sternberg, M. J. E. The Phyre2 web portal for protein modeling, prediction and analysis. *Nat Protoc.* **10,** 845–858 (2015).
2. DeLaBarre, B. & Brunger, A. T. Complete structure of p97/valosin-containing protein reveals communication between nucleotide domains. *Nat. Struct. Biol.* **10,** 856–863 (2003).
3. Kabsch, W. & Sander, C. Dictionary of protein secondary structure: Pattern recognition of hydrogen-bonded and geometrical features. *Biopolymers* **22,** 2577–2637 (1983).
4. Loibl, M. *et al.* The drug diazaborine blocks ribosome biogenesis by inhibiting the AAA-ATPase Drg1. *J. Biol. Chem.* **289,** 3913–3922 (2014).
5. Kappel, L. *et al.* Rlp24 activates the AAA-ATPase Drg1 to initiate cytoplasmic pre-60S maturation. *J. Cell Biol.* **199,** 771–782 (2012).
6. Pertschy, B. *et al.* Cytoplasmic recycling of 60S preribosomal factors depends on the AAA protein Drg1. *Mol. Cell. Biol.* **27,** 6581–6592 (2007).
7. Zakalskiy, A. *et al.* Structural and enzymatic properties of the AAA protein Drg1p from Saccharomyces cerevisiae. Decoupling of intracellular function from ATPase activity and hexamerization. *J. Biol. Chem.* **277,** 26788–26795 (2002).
